# Spectroscopic Determination of Fluoride Using Eriochrome Black T (EBT) as a Spectrophotometric Reagent from Groundwater

**DOI:** 10.1155/2021/2045491

**Published:** 2021-12-28

**Authors:** Atinafu G/Mariam, Abebe Diro, Tsegaye Girma Asere, Demelash Jado, Fekadu Melak

**Affiliations:** College of Natural Sciences, Department of Chemistry, Jimma University, Jimma, Ethiopia

## Abstract

Fluoride health problem is a great concern worldwide, most often as a result of groundwater intake. Thus, determination of fluoride is vital to take appropriate measures upon fluoride contamination of water. Potentiometric method of analysis is reliable for the determination of fluoride in various samples. In addition, spectroscopic methods are found important to quantify fluoride levels from water; however, several factors hinder its easier determination. Among the bottlenecks, the use of toxic chemicals and tedious steps in preparing chemicals (e.g., SPADNS method) are to mention a few. In this study, a spectrophotometric method was developed for the determination of fluoride from groundwater using Eriochrome Black T (EBT) as a spectroscopic reagent. Experimental parameters that influence the determination of fluoride including ligand type, kinetics, pH, and ligand-to-metal ratio were assayed. Evaluation of fluoride levels showed that Beer–Lambert's law is obeyed in the range of 0.3–5.0 mg/L at 544 nm. The calibration curve, resulting in good linearity (*R*^2^ = 0.9997), was considered during quantitative analysis of the samples and in the spiking analysis. The limits of detection (LOD) and quantification (LOQ) of the method were found to be 0.19 and 0.64 mg/L, respectively. The precision studied in terms of intraday and interday at three concentration levels showed less than 5.4% RSD. Applicability of the method was investigated by analyzing groundwater samples spiked with fluoride standards, and satisfactory recoveries in the range of 98.18–111.4 were demonstrated. The developed spectrophotometric method has been successfully applied for fluoride determinations in groundwater samples. Thus, it could be used as an attractive alternative for the determination of fluoride from groundwater.

## 1. Introduction

Fluoride is a common groundwater pollutant, mostly from geogenic sources. It is widely distributed in the Earth's crust existing in several mineral forms such as fluorite (CaF_2_), cryolite (Na_3_AlF_6_), monofluorophosphate (PO_3_F^2−^), and fluoroapatite (Ca_5_(PO_4_)_3_F) [1]. Fluoride is used in a number of applications including dental care [2], agrochemical use [3], pharmaceuticals [4], water fluoridation [5, 6], and even in chemical industries including nuclear reactors [7, 8]. Fluoride leaching in water bodies is dependent on mineralogical chemistry, well depth, hydrologic conditions, and geologic structures, being calcium-poor aquifers expected to possess an enhanced level of fluoride [9].

Human exposure to fluoride occurs from various sources including food, drug, cosmetics, and drinking water being the major source of daily intake [10]. It has been shown to cause a significant impact on human health when excessive fluoride via drinking water occurs, including dental fluorosis and skeletal fluorosis [11, 12]. The World Health Organization (WHO) has set a maximum contamination limit value of 1.5 mg/L for fluoride in drinking water to prevent health risks [13]. In fact, a low level (0.5–1.5 mg/L) of fluoride is assumed to be an essential component of human health in preventing dental caries [14, 15]. As a result, when fluoride sources are negligible, pharmaceutical preparations including prescription drops, lozenges, or tablets and fluoridated toothpastes are recommended [16].

Several techniques have been applied in the determination of fluoride from various matrices. Fluoride analysis techniques including electrochemical, chromatographic, titrimetric, capillary zone electrophoresis, fluorescence sensing, and spectroscopic methods have been developed [16–20]. Ion chromatography is expensive; ion-selective method is economical and sensitive in relation to UV-Vis spectroscopy. Ion-selective electrode method is widely used and suitable for industrial and field monitoring [21]; however, there are several disadvantages associated with it. It requires advanced cautions and buffer systems that require extra care in steps of chemical preparation. Buffer system during fluoride analysis using the ion-selective electrode involves citrate, cyclohexanediaminetetraacetic acid (CDTA), total ionic strength adjustment buffer (TISAB), diethylenetriamine pentaacetate (DTPA), and ethylenediaminetetraacetic acid (EDTA) [16, 22, 23]. The effect of coexisting ions in water that form complexes with fluoride has been another challenge in potentiometric/ion-selective determination of fluoride [24].

Spectroscopic determination of fluoride using chromophore-based reagents has been extensively studied. For instance, the use of complexes of flavonoid chrysin [25], aluminum complexes of triphenylmethane dyes [18, 26], interaction of fluoride with Al(III)-salicylic aldehyde acylhydrazones [18], reaction with zirconium ions and SPADNS [27], coordination of Al(III) with Alizarin Red-S [28], Al-xylenol orange complex [29], and aluminum quinalizarin complex [30] are to mention a few fluoride extraction ways. In fact, the use of the aforementioned ligands would help in enhancing sensitivity and selectivity of fluoride extraction for the spectrophotometric determination. Similar phenomena have been enacted in the spectrophotometric determination of silver(I), copper(II), and cobalt(II) [31–33]. However, methods such as SPADNS possess lengthy solution preparations and use toxic chemicals. Therefore, a relatively simple, easier method of fluoride determination using a common indicator (EBT) has been developed in this research. Eriochrome Black T is among essential azo dyes used as a common indicator in complexometric titrations for the estimation of various metal cations such as Ca^2+^, Mg^2+^, and Zn^2+^ ions [34, 35]. A reactive azo dye such as EBT contains one or more azo bonds (-N=N–) that act as a chromophore. The visible spectra of azo dyes (e.g., EBT) exhibit intense bands at 510–560 nm. The spectra of azo dye to M(III) complexes are very similar to those of free dyes except changes of about 10–30 nm bathochromic shift in its absorption maxima [36].

Accordingly, this study was carried out to develop a simple spectrophotometric method for the determination of fluoride from groundwater using EBT as spectrophotometric reagent. Thus, parameters including metal-to-ligand ratio, pH effect, interference study, stoichiometry of the Al-EBT complex with fluoride, and real sample analysis have been explored.

## 2. Materials and Methods

### 2.1. Reagents

Chemicals used in the experiment include 99.0% NaF (Finkem, India), Eriochrome Black T (Merck, Germany), glacial acetic acid (Sigma-Aldrich, Germany), sodium acetate buffer, 37% HCl (Fisher Chemicals, UK), and ammonia solution (Merck, Germany). Sodium chloride (Fisher Chemicals, UK), 69% HNO_3_ (Fine Chemical Industries, India), hydrated aluminum nitrate Al(NO_3_)_3_.9H_2_O (Nice Chemicals Ltd., India), sodium sulphate (Blulux Laboratory reagent, India), and hydrated aluminum chloride (AlCl_3_.6H_2_O) were used during the study. Eriochrome Black T (EBT) was also used in total hardness determination besides its use as a ligand in fluoride determination. A 1,10-phenanthroline method was used to determine iron in well water that is used to test the method in real groundwater sample analysis. In this method, concentrated HCl, hydroxylamine, acetate buffer, and 1,10-phenanthroline were employed. Nitrate was measured using a UV-Vis spectrophotometer (Analytik Jena Specord 200, Germany). H_2_SO_4_ (titrant), phenolphthalein and bromocresol green indicators, and calcon indicator were used to determine both calcium hardness and alkalinity.

### 2.2. Instruments and Apparatuses

A double-beam spectrometer (Analytik Jena, Germany) was used to measure the absorbance of the sample. pH meter (Bante Instruments, pH 902, USA) equipped with the electrode was used to measure the pH values of sample solutions. Quartz cuvette (1 cm), micropipettes (10–100 *μ*L and 100–1000 *μ*L) (Dragonmed, Shanghai, China), and filter paper (Whatman No. 542, England) were utilized during the experiment.

### 2.3. Solution Preparation

1000 mg/L fluoride stock solution was prepared by dissolving 2.21 g of dried sodium fluoride in 1 L of deionized water. The stock solution was kept in a refrigerator using plastic bottles until it was used. A range of calibration standards were prepared by serial dilution to determine fluoride levels at each parameter optimization. 1.0 × 10^−3^ M Al^+3^ and 1.0 × 10^−3^ M of EBT were prepared from 0.1 g of Al(NO_3_)_3_.9H_2_O and 0.12 g EBT, respectively.

#### 2.3.1. Preparation of the Al-EBT Complex

The Al-EBT complex was prepared by mixing 1 : 1 ratio of aluminum to EBT, 1.0 × 10^−3^ M of aluminum and 1.0 × 10^−3^ M of EBT in 250 mL deionized water, and diluted to 5.0 × 10^−5^ M for the spectrophotometric measurements.

### 2.4. Reaction of Fluoride with the Prepared Complex

To optimize the stoichiometry of the reaction mixture, EBT-Al with fluoride, variable concentration of the prepared Al-EBT complex and fluoride was considered, making the concentration of fluoride constant on the one hand. Also, experiments were performed by varying the concentration of fluoride with constant concentration of the complex, Al-EBT, on the other hand. The formation of the product, EBT-Al-F, was confirmed by wine red color observation at the acidic medium.

### 2.5. Dye Selection

The dyes including methylene orange, brilliant green, bromocresol green, methyl violet, malachite green, and Eriochrome Black T were studied to select appropriate ligand for the fluoride determination. This is to find stable complexes of aluminum to ligand that obey the Beer–Lambert law when the reaction is complete with fluoride. Trial tests for each of the dyes have been made (figure not shown).

### 2.6. Reaction Time

Reaction time is among the important parameters that affects the reaction and can influence quantitative evaluation of reaction products [37]. Equilibrium time is essential to quantify the analyte and hence examined during the reaction progress. The stability of the complex in water was examined by measuring the absorbance of the prepared admixture (fluoride with the Al-EBT complex) solution at different time intervals, starting from initial 15 min to 48 hr.

### 2.7. Effect of pH

The influence of pH on the formation of the complex was studied by carrying out the reaction in sodium acetate/acetic acid buffer solution of pH ranging from 4.0 to 5.4.

### 2.8. Stoichiometry of the Al-EBT Complex with Fluoride

The stoichiometric ratio between aluminum and EBT was evaluated by Job's method of continuous variation [38]. In this method, solutions of aluminum and EBT with identical molar concentrations (5.0 × 10^−5^ M) were mixed in varying volume ratios (1 + 9 to 9 + 1 mL) in such a way that the total volume of each mixture was the same which is 10 mL. The absorbance of the solutions was measured at a wavelength of 544 nm, and mole fraction was calculated by using the following formula. For the reaction, *M* + nL⟶ML_n_, the composition of the complex was given by the volume fraction ratio as follows:

 
*n*=*x*/1 − *x*, where *n* is the subscript of the ligand and
 
*X*: [1 − *X*], where *X* =  *V*_*m*_/*V*_*m*_+*V*_*L*_ and *V*_*M*_ = volume of metal and *V*_*L*_ = volume of the ligand

### 2.9. Method Validation

Method validation is the process of proving (through scientific studies) that an analytical method is acceptable for its intended use. It is used to ensure confidence in the analytical data throughout product development. The validation was carried out in terms of linearity, the limit of detection, the limit of quantification, precision, and accuracy studies [39, 40].

#### 2.9.1. Limit of Detection (LOD)

LOD is the lowest concentration or mass of species under investigation in a sample that can be detected with a specified level of confidence but not necessarily quantified, under stated condition of the test. It was evaluated from LOD = 3.3*σ*/*S*, where *σ* = the standard deviation of *y*-intercepts of regression lines and *S* = the slope of the calibration curve.

#### 2.9.2. Limit of Quantification (LOQ)

LOQ is the lowest amount of analyte in a sample which can be quantitatively determined with a suitable precision and accuracy. LOQ = 10*σ*/*S,* where *σ* = the standard deviation of *y*-intercepts of regression lines and *S* = the slope of the calibration curve.

#### 2.9.3. Precision

Precision can be determined by repeatability (intraday), intermediate precision (interday), and reproducibility. Repeatability is a measure to express precision obtained with the same method, on the same test material, in the same laboratory, by the same operator, and using the same equipment within short intervals of time. Thus, in this research, repeatability was evaluated assaying three level determinations with the same concentration, during the same day, under the same experimental conditions in the morning session and afternoon session. Intermediate precision was analyzed by comparing the assays in three determinations at the same concentration within five different days and expressed as relative standard deviation (RSD).

#### 2.9.4. Accuracy

Accuracy is an agreement between measured and real values. The accuracy of the proposed method was evaluated using recovery studies after spiking standard fluoride on a known amount of analyte of interest. The spiking has been made on a real groundwater sample, initially well characterized for its composition. Three different solutions of fluoride were prepared for each concentration level (0.5, 1, and 1.5 mg/L), and the accuracy was calculated on the basis of percentage recovery. The percentage recovery values were calculated by comparing the amount obtained from the spiked samples with the amount of fluoride in which the concentration was actually added, as follows:

(1) $$\begin{array}{c} \%\text{Recovery}=\frac{C_{\text{spiked}}-C_{\text{unspiked}}}{C_{\text{added}}}*100, \end{array}$$

where *C* is the amount of the sample.

### 2.10. Interference Study

The interference studies were evaluated by measuring the influence of anions such as chloride, nitrate, and sulphate on the determination of 1.5 mg/L fluoride. Therefore, the expected interfering anions were added in such concentrations of 2–300, 25–500, and 40–700 mg/L for nitrate, sulphate, and chloride, respectively.

### 2.11. Determination of Fluoride in a Real Groundwater Sample

The method under investigation was tested using a real groundwater sample by appropriate dilution. The water sample was diluted, and then spike analysis was performed upon it. Fluoride was analyzed using a double-beam UV-Vis spectrophotometer (Analytik Jena Specord 200, Germany). All experiments, unless mentioned, were performed in triplicate, and the average results were reported together with error bars based on standard deviation of the triplicate measurements.

## 3. Results and Discussion

### 3.1. Optimization of Parameters

#### 3.1.1. Dye Selection

In previous works, several dyes have been tested for the determination of fluoride including zirconium-xylenol orange, m-cresol red, crystal violet, leucomalachite green, bromocresol purple, brilliant blue R, Patent blue VF sodium salt, acid violet, Alphazurine A, parafuchsin, Victoria blue R, ethyl violet, light green, bromocresol green, malachite green oxalate lissamine green B, pyrocatechol violet, aluminon, chromeazurol B, and malachite green carbinol base [26, 41, 42]. However, only few work well in the determination of fluoride by complexing with M(III), most often aluminum ion. In this work as well, dyes as a ligand for the determination of fluoride including methylene orange, brilliant green, bromocresol green, methyl violet, and Eriochrome Black T have been tried. However, most of them fail to be sensitive and stable for analysis. Due to the sensitive absorption maxima of the dye and its aluminum complex to conform Beer–Lambert's law for certain concentration ranges, EBT was considered for further analysis. The ionic nature of the dye could be one possible factor to influence the complex formation. EBT is an anionic dye and can react with cation aluminum to form Al-EBT complex. Azo dyes such as EBT are reactive with one or more azo bonds (–N=N–) that act as a chromophore in the molecule [43].

#### 3.1.2. Selection of Maximum Wavelength

**Figure 1:**
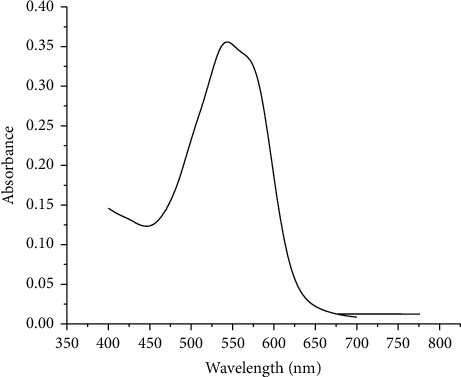
UV-Vis spectrum of the Al-EBT-F complex in water at 5 × 10^−5^ M concentration.

The spectra of EBT alone showed a maximum absorbance at 535 nm, whereas the ternary mixtures of Al-EBT-F, as shown in Figure 1, were observed at 544 nm, which is a working wavelength in the determination of fluoride. Bathochromic shift has been observed because of the displacement of the naphthalic hydrogens during the chelation process. It is known that deprotonation of phenols is accompanied by a bathochromic shift of the absorption band [44, 45].

#### 3.1.3. Reaction Condition of Fluoride with the Al-EBT Complex

**Figure 2:**
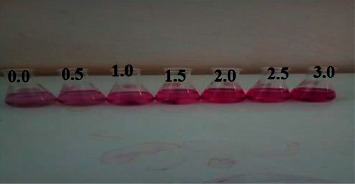
Color intensifications upon fluoride level increments on the addition of different amounts of fluoride from 0.5 to 3.0 mg/L to 5.0 × 10^–5^ M metal-EBT 1 : 1 complex.

**Figure 3:**
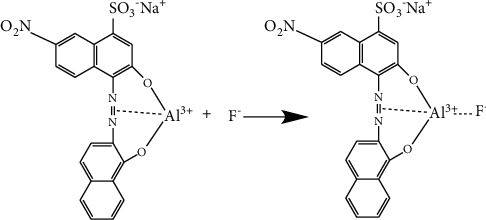
Proposed mechanism of the chemical reaction of Al-EBT with fluoride.

The absorption spectra of the reaction of fluoride with the Al-EBT complex showed an increase in intensity of the color of the complex as the concentration of fluoride increased. The phenomena could indicate fluoride interaction and formation of ternary complexes. Wine red colored EBT-Al reacts with F^−^ and gives intense wine red color as is demonstrated in Figure 2, suggesting interaction of F^−^ with the complex. The reaction of EBT-Al with F^−^ is somehow stabilizing phenomena to a certain extent because the color of the EBT-Al complex remains more intensified as fluoride was added. The mechanism reaction is proposed in a scheme in Figure 3, instead of substitution reactions. Similar results have been observed for the direct determination of fluoride [30, 46–48]. In contrary to this fact, some literature studies with ligands other than EBT reported that adding fluoride into aluminum complexes showed fading up of the color to light, enabling indirect measurement of fluoride from water [20, 40].

#### 3.1.4. Optimization of Reaction Time

**Figure 4:**
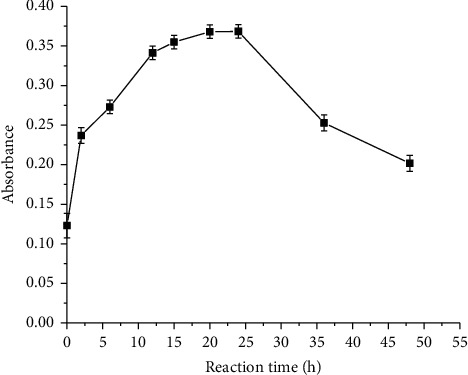
Effect of reaction time on the determination of fluoride by EBT as a spectrophotometric reagent (conditions: pH = 5.0 and concentration of aluminum and EBT: 5.0 × 10^−5^ M).

Reaction time is an important parameter to influence reaction products. The process of formation of the Al-EBT-F^−^ complex in aqueous solutions has been studied and presented as a function of absorbance enhancement in Figure 4. When the reaction duration increased until 20 h, the amount of the ternary complex became more intense, providing higher absorbance that indicates higher concentration of the product at fixed reaction conditions. However, after 20 h, the reaction remained constant for about four hours. Thus, reaction time for the fluoride determination has been performed considering 24 h as an equilibrium and stable condition to prove Beer–Lambert's equation. In fact, possibilities of fluoride analysis from 20 h to 24 h are proven. A longer reaction duration of 24 h afterwards revealed the decrement in absorbance violating Beer–Lambert's assumption.

#### 3.1.5. Optimization of pH

**Figure 5:**
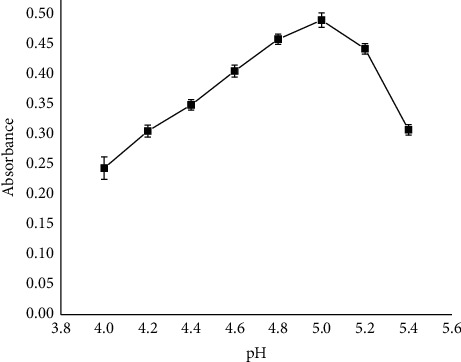
pH effect on the determination of fluoride using EBT as a spectrophotometric reagent (conditions: concentration of aluminum and EBT: 5.0 × 10^−5^ M and reaction time: 24 h).

The influence of pH upon reaction of fluoride with the Al-EBT complex is investigated and presented in Figure 5. Higher pH values may inhibit the reaction of fluoride with Al-EBT complexes. Possible reasons could be the increase in the concentration of OH^−^ that has similar electrical charge and radius with fluoride. Thus, pH optimization overwhelmed in acidic media from 4 to 5.4, and a maximum absorbance was found to be at pH 5.0, preferred for further study. At pH below 4.5, the UV-Vis might be lower than the actual concentration of the fluoride ions.

#### 3.1.6. Molar Ratio of the Metal and EBT Complex

**Figure 6:**
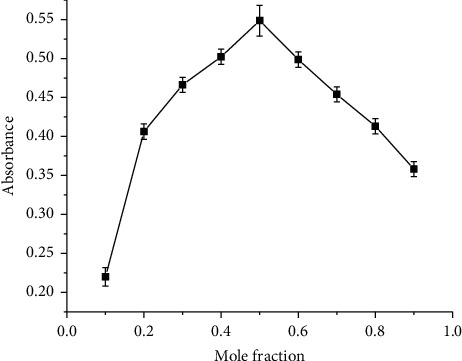
The graph of continuous variation method plotted as absorbance versus mole fraction of complexes of 5.0 × 10^−5^ M aluminum to EBT.

The results obtained from applying Job's method of continuous variation indicated that aluminum and EBT can form 1 : 1 complex because the maximum absorbance appeared at *x* = 0.5 as shown in Figure 6. The complex is plausibly due to the lone pair of electrons on the oxygen atom in Eriochrome Black T delocalized into the outer orbitals of the Al^3+^ ions. The proposed complex has a Π-bond, between the donor (oxygen atom) and the acceptor (Al^3+^ ion), which increased the binding energy of the central Al atom. Aluminum : Eriochrome Black T, 1 : 1 complex, is brown in water and changes its color to light red, getting intense with an increased concentration of fluoride. This could indicate the probable formation of the EBT-Al-F^−^ complex as a mechanism of determination.

#### 3.1.7. Interference Study

**Figure 7:**
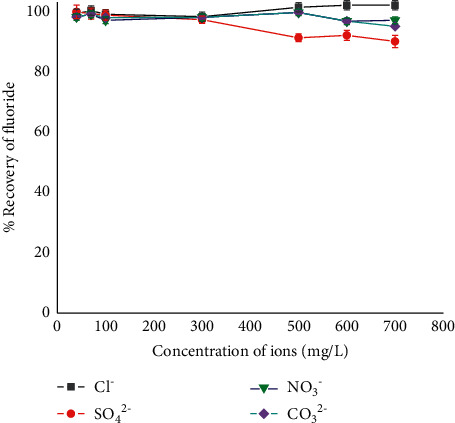
Effect of various amounts of interfering anions on 5.0 × 10^−5^ M Al-EBT complex plus 1.5 mg/L fluoride at 544 nm, with conditions of reaction time of 24 h and pH 5.0.

The interference studies were evaluated by measuring the influence of anions such as chloride, nitrate, and sulphate upon 1.5 mg/L fluoride solution during determination. The results of interference study are summarized in Figure 7. Therefore, the expected interfering anions were added in such concentrations for nitrate, sulphate, and chloride in the range of 2–300, 25–500, and 40–700 mg/L, respectively. From the studied ions, SO_4_^2−^ influenced the absorbance of the target analyte at higher concentrations. The percent recovery of fluoride determined in the presence of interference ions was calculated as in the following equation:

(2) $$\begin{array}{c} \text{recovery}\left(\%\right)=\frac{\text{absorbance of the interfering ion}}{\text{absorbance of the analyte}}\times 100. \end{array}$$

The data showed that chloride and nitrate did not interfere with the determination of fluoride, whereas sulphate showed to interfere as it is commonly reported to inhibit fluoride detection as in most visual and photometric methods [49, 50]. This might be due to the competition of SO_4_^2−^ with fluoride to form a complex with the metal. In the present work, when the amount of sulphate is higher than 200 mg/L, it showed to interfere with the determination of fluoride. However, sulphate usually presents at lower concentration levels than 200 mg/L in Ethiopian groundwaters, which makes the method to work in real groundwaters with less interference from sulphate.

### 3.2. Determination of Fluoride

#### 3.2.1. Calibration Curve

**Figure 8:**
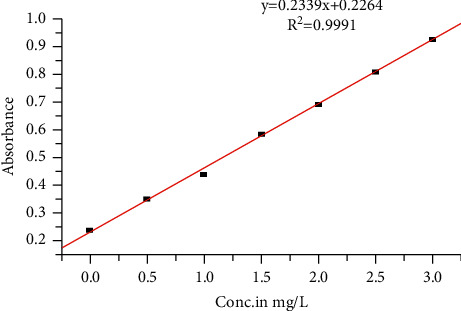
Calibration curve for the determination of fluoride at 544 nm.

The calibration curve was drawn using series of concentrations of fluoride in the range of 0.5, 1.0, 1.5, 2.0, 2.5, and 3.0 mg/L. This was obtained at optimum conditions of pH 5.0 and reaction duration of 24 h. Each concentration level was read in triplicate, and average values were taken. Then, standard calibration curve was plotted from the absorbance values obtained for the five standards of fluoride solutions by taking concentration (mg/L) on the *x*-axis and absorbance values on the *y*-axis. The linear regression equation was found to be *y* = 0.2339*x* + 0.2264 and *R*^2^ = 0.9991, which is satisfactory for quantitative analysis of the analyte in a proposed groundwater sample as shown in Figure 8.

#### 3.2.2. Precision Study

The precision of the proposed spectrophotometric method was investigated in terms of intraday precision (repeatability) and interday precision (within-lab reproducibility). Intraday and interday precisions were studied by preparing three different concentration levels (0.5, 1.5, and 2.5 mg/L) of fluoride. For the case of intraday precision study, each concentration level was analyzed in triplicate on the same day (in morning and afternoon sessions). Under the same experimental conditions, interday precision of the method was evaluated at three concentration levels earlier used for intraday precision studies, for five consecutive days with the same time interval. The relative standard deviation (RSD) of the intraday (repeatability) and interday (within-lab reproducibility) precisions of the proposed method is presented in Table 1. The %RSD of both intra- and interday precisions of the three concentration levels was found below 9.00%, which indicated that the developed method showed reasonable acceptable range for the analysis of the target analyte.

### 3.3. Analysis of the Real Sample and Recovery Studies

The applicability of the developed method was evaluated by performing relative recovery studies utilizing three level standards spiked upon real groundwater samples. In order to demonstrate the applicability and reliability of the method for real samples, three samples of groundwater were prepared and analyzed by the method. For each level, blank samples (unspiked) were also analyzed by the same method. The %RR of the target analyte for each level is demonstrated in Table 2. The relative recovery (%RR) was calculated as in the following equation:

(3) $$\begin{array}{c} \%\text{RR}=\frac{C_{\text{spiked}}-C_{\text{unspiked}}}{C_{\text{added}}}\times 100, \end{array}$$

where *C* is the concentration of a sample.

The observed percent recovery of the target analyte in groundwater samples ranged from 98.18 to 111.40, which briefly indicated that the proposed method had acceptable relative recoveries for the determination of fluoride from groundwater samples. The physicochemical parameters of groundwater samples (well water ≠1) employed in the experiment are analyzed and presented in Table 3.

### 3.4. Comparison of Methods in Fluoride Detection

The developed method for the determination of fluoride has been compared with related analytical techniques reported in the literature. The comparison is made in terms of linear range, limit of detection (LOD), and limit of quantification (LOQ) and given in Table 4.

As it can be seen from Table 4, it was noted that the method developed has provided better linearity and similar or better LOD and LOQ compared with reported literature studies. Thus, the method can be considered as an attractive alternative and less toxic for determining fluoride in groundwater samples.

## 4. Conclusion

The analytical method for the measurement of fluoride with appropriate ligand (EBT) is presented here. EBT was well suited for the spectrophotometric reagent of fluoride analysis. Promising results were obtained with 1 : 1 ratio of Al and EBT complex. This complex was used as a spectrophotometric reagent for fluoride determination in the range of 0.3–5.0 mg/L. The obtained results demonstrated that the developed method was fully functional with 0.19 mg/L detection limit and 0.64 mg/L quantification limit. The method showed promising applications in real groundwater samples, providing 98.18 to 111.40% recoveries.

## Figures and Tables

**Table 1:** Reproducibility and intraday and interday precisions during fluoride determination.

Levels of F^−^ in mg/L	Precision	
	Intraday (%RSD)	Interday (%RSD)
0.5	4.91	2.51
1.5	6.94	7.30
2.5	6.75	8.18

**Table 2:** Analytical results of groundwater sample analysis via spiking (*n* = 3).

Sample type	Fluoride standard spiked (mg/L)	This method, F^−^ found (mg/L) in sample waters after dilution^a^	Fluoride found (spiked + sample water)^b^	% Recovery	RSD, %
GW-1	0.25	2.50	2.70	98.18	3.20
GW-1	0.5	2.50	3.06	111.40	6.20
GW-1	1.5	2.50	2.86	110.60	4.50

^a^Fluoride found in groundwater after 7-fold dilution. ^b^Total fluoride in 7-fold-diluted groundwater plus spiked standard.

**Table 3:** Physicochemical parameters of the well water ≠1 used in the experiment.

Parameters	Concentration
pH	6.77
Turbidity, NTU	7.45
TDS, mg/L	215
Chloride, mg/L	4.6
Nitrate, mg/L	0.70
Total hardness, mg/L	36
Iron, mg/L	0.012
Magnesium, mg/L	16.90
Calcium, mg/L	21.50
Conductivity, *μ*S/cm	95.55
Alkalinity (by bicarbonate), mg/L	17
Fluoride, mg/L	17.51

**Table 4:** Comparison method validation parameters with previously reported methods.

Ligands used in spectroscopic analysis of fluoride	Linear range (mg/L)	LOD (mg/L)	LOQ (mg/L)	Reference
Al to quinalizarin	0.5–2.0	0.1	0.3	[67]
Al to resorcin blue	0.2–1.0	0.2	0.5	[72]
Al to flavonoid chrysin	0.5–3.0	0.1	0.3	[68]
Al to malachite green	0.5–4.0	0.1	0.3	[71]
Al to chromeazurol B	0.5–4.0	0.2	0.5	[71]
Zr to resorcin blue	0.1–4.0	0.07	0.2	[87]
Al to EBT	0.3–5.0	0.19	0.64	This work

## Data Availability

The data supporting the results of our study are available from the corresponding author upon request.
